# Knowledge, Attitudes, and Practices of Antibiotic Use and Antimicrobial Resistance Among Medical Students in Southern Iraq

**DOI:** 10.7759/cureus.100550

**Published:** 2026-01-01

**Authors:** Afnan S Al-Maliki, Ridha A Al Mohammed, Jawad K Albazoony

**Affiliations:** 1 Department of Pharmacology, College of Medicine, University of Basrah, Basra, IRQ

**Keywords:** antibiotics stewardship, antimicrobial resistance (amr), inappropriate antibiotic use, iraq, kap study, medical students

## Abstract

Background

Antimicrobial resistance (AMR) is a growing global public health threat, largely driven by inappropriate antibiotic use. Low- and middle-income countries are disproportionately affected, yet data on AMR-related knowledge and practices among medical students in Iraq remain limited. Evaluating medical students’ knowledge, attitudes, and practices regarding antibiotic use is essential for informing effective educational and stewardship interventions.

Methodology

This descriptive cross-sectional study was conducted among medical students at the University of Basrah during the 2024-2025 academic year using a questionnaire developed after a literature review of previously published KAP studies. A stratified sampling method was applied. Data were analyzed using descriptive statistics, Chi-square tests, independent samples t-tests, ANOVA, and Kruskal-Wallis tests with IBM SPSS Statistics for Windows, Version 26 (Released 2018; IBM Corp., Armonk, New York, United States).

Results

A total of 600 students completed the survey, of which 51.7% were female. Knowledge scores improved significantly with each academic year (p<0.001), yet 75.9% showed poor practice, while the total proportion of students that demonstrated poor knowledge was 51.0%. Findings also indicate a general trend towards a more positive attitude, from 20.9% of second-year medical students demonstrating a positive attitude to 46.5% of sixth-year students. Interestingly, a gap was noted between students’ knowledge levels and actual antibiotic use practices.

Conclusion

Despite the significant improvement in knowledge levels, poor practices persisted among medical students, highlighting the need for targeted interventions to raise awareness and improve practices among future prescribing physicians.

## Introduction

While antibiotics have been an essential part of treating and preventing bacterial infections since their discovery in 1928 [1], their effectiveness has been decreasing, leading to an urgent public health crisis and claiming approximately one million lives each year since 1990 [2,3]. The reason behind this is the growing problem of antimicrobial resistance (AMR), which occurs due to induced selection pressure associated with excessive and inappropriate use [4]. The estimated mortality associated with AMR was 4.71 million in 2021 [5]. This is especially prevalent in low- to middle-income countries, which bear a larger share of AMR-associated morbidity and mortality [6]. Major contributors to this behavior among physicians include overly permissive policies [7,8], patient and family pressure [9], lack of public awareness [10], self-medication [11], lack of rapid tests [12], and limited knowledge of the importance of AMR among prescribing physicians [13]. A cross-sectional survey in Iran showed that the majority of Iranian physicians had poor knowledge of AMR and antimicrobial stewardship programs. Moreover, only 65.9% of them showed proper practice [14]. Similar findings have been reported among physicians in Turkey [15], Saudi Arabia [16], Oman [17], and Jordan [18]. In Iraq, antibiotic misuse is prevalent, and AMR is a serious and growing health risk [19]. Despite the risks associated with the spread of AMR, the Ministry of Health in Iraq still allows the sale of certain antibiotics without the need for a prescription [7].

Medical students and interns play an important role in combating AMR, as they represent future prescribers whose practices will shape antibiotic use patterns. Assessing their knowledge, attitudes, and practices (KAP) regarding antibiotic use can help inform targeted educational interventions. Surveys conducted among medical students in the region have revealed persistent gaps in knowledge, high rates of self-medication, and poor antibiotic use practices [20].

This study aims to fill this gap by assessing medical students’ knowledge, attitudes, and practices related to antibiotic use at the University of Basrah, so that gaps and misconceptions can be addressed through education and contribute to Iraq’s wider efforts against AMR.

## Materials and methods

Study design

This was a descriptive cross-sectional study conducted during the 2024-2025 academic year to assess knowledge, attitudes, and practices related to antibiotic use and resistance among medical students at the College of Medicine, University of Basrah.

Study setting

The study was conducted on the campus of the College of Medicine, University of Basrah.

Sample size and data collection

A total of 612 medical students participated in the study. A stratified sampling method was applied, targeting at least 20% of students from each academic year (second through sixth year) to ensure proportional representation. Enrollment numbers were obtained from the Student Affairs Department and included 700 second-year, 548 third-year, 582 fourth-year, 778 fifth-year, and 377 sixth-year students. Data collection was conducted in lecture halls, college laboratories, and student common areas. The study objectives were explained, and printed self-administered questionnaires were distributed to participating students. Students at the institution are familiar with peer-led academic research activities, which facilitated cooperation and attainment of the targeted sample. Participation was voluntary, with no incentives offered and no academic consequences. Inclusion criteria comprised all second- to sixth-year medical students who provided informed consent. Exclusion criteria included first-year students, as they had not commenced their academic year at the time of data collection, and students who declined participation.

Study tool

Data were collected using a questionnaire adapted from previously published, peer-reviewed KAP studies assessing antibiotic use and AMR [21-23] and further refined by the research team following a review of relevant literature. Faculty members from the Department of Pharmacology reviewed the instrument for accuracy and appropriateness. The questionnaire was pilot-tested on 20 participants to assess clarity and feasibility, and minor wording revisions were made based on participant feedback; pilot data were excluded from the final analysis. The final questionnaire consisted of four sections: demographics (four questions), knowledge (five questions), attitudes (seven questions), and practices (six questions). Smoking status was included to provide a more comprehensive description of the study population. Knowledge items were multiple-choice, while attitude and practice items used three-point Likert scales with agree/disagree/not sure and yes/no/sometimes response options, respectively [24].

The overall Cronbach’s alpha was low (α = 0.43), which was expected given the exploratory, descriptive design of the questionnaire, the limited number of items per domain, and the use of a 3-point Likert scale [25].

Data analysis

Questionnaire responses were entered directly into IBM SPSS Statistics for Windows, Version 26 (Released 2018; IBM Corp., Armonk, New York, United States) for data coding and statistical analysis, while Microsoft Excel (Microsoft Corporation, Redmond, USA) was used only for table formatting. Categorical variables, including gender, academic year, smoking status, family background in healthcare, and KAP levels, were summarized using frequencies and percentages and presented in tables, while continuous variables, including total knowledge, attitude, and practice scores, were described using means and standard deviations. The chi-square test was applied to categorical variables and one-way analysis of variance (ANOVA) was applied to compare mean scores across academic years, with statistical significance set at p < 0.05. The Kruskal-Wallis test was used as a non-parametric alternative where appropriate.

The scoring system and scales were developed by the research team, inspired by the modified Bloom’s cutoff, where scores below 50% were considered poor or negative. Higher score ranges were adjusted to fit the structure of this questionnaire. No copyrighted or licensed instruments were utilized. Score categorization was applied to facilitate descriptive interpretation and comparison across academic years and was not intended to represent standardized or diagnostic thresholds.

Ethical approval

Ethical approval was obtained from the Department of Pharmacology, College of Medicine, University of Basrah (Ref. No. 22), on October 22, 2024, prior to the commencement of data collection. Consent was obtained from each participant before receiving a copy of the questionnaire. No identifiers were collected, and anonymity was preserved. Artificial intelligence (AI) tools were used only for language editing and formatting. No AI systems were involved in data analysis, interpretation, or the generation of scientific content.

## Results

Respondents' characteristics

A total of 612 students initially participated in the study. After reviewing the returned questionnaires, 12 were excluded due to incomplete or missing responses, resulting in a response rate of 98%. Of the remaining 600 students, participants were distributed across academic years from second to sixth year, with a nearly even gender distribution. Approximately one-third reported having a family background in healthcare, and most participants were non-smokers (Table 1).

**Table 1:** Demographic characteristics of participants

Variables	Frequency (N)	Percent (%)
Gender		
Male	287	47.8
Female	310	51.7
Academic Year		
Second	141	23.5
Third	109	18.2
Fourth	116	19.3
Fifth	159	26.5
Sixth	75	12.5
Smoking		
Yes	78	13.0
No	478	79.7
Family Member in Healthcare		
Yes	219	36.5
No	375	62.5

Analysis of knowledge scores and levels

Good knowledge was defined as a score of five out of five points, adequate knowledge as three to four points, and poor knowledge as zero to two points. The overall mean knowledge score of participants was 3.92 ± 1.37. A Kruskal-Wallis test was conducted as a non-parametric alternative to ANOVA to compare knowledge scores across academic years. The test revealed a statistically significant difference in knowledge scores among the five academic years (H(4) = 173.21, p < 0.001), with mean ranks increasing from second- to sixth-year students. Responses to individual knowledge questions are detailed in Tables 2, 3. Sub-analyses of knowledge levels were conducted according to gender and academic year. No statistically significant difference in knowledge levels was observed between male and female participants (χ²(2) = 2.117, p = 0.347). Analysis of the relationship between knowledge levels and academic year showed a statistically significant association (χ²(8) = 164.671, p < 0.001) (Table 4).

**Table 2:** Responses to knowledge-related questions on antibiotic use and antimicrobial resistance among medical students n: frequency; %: percentage

Questions Evaluating Knowledge	True		False		Not sure		Total
	n	%	n	%	n	%	n
Unjustified use of antibiotics can lead to bacterial resistance	467	77.80%	40	6.66%	93	15.50%	600
Antibiotics are effective against viruses	115	19.20%	367	61.20%	118	19.70%	600
Misuse of antibiotics can result in future failure of treatments	491	81.80%	26	4.30%	83	13.80%	600
Bacterial resistance is an important global issue	525	87.50%	16	2.70%	59	9.80%	600

**Table 3:** Responses to the question assessing knowledge of the cause of the common cold and flu among medical students n: frequency; %: percentage

Questions Evaluating Knowledge	Bacteria		Virus		Not sure		Total
	n	%	n	%	n	%	n
What is the most common cause of common cold/flu?	35	5.80%	503	83.80%	62	10.30%	600

**Table 4:** Distribution of knowledge levels related to antibiotic use and antimicrobial resistance across academic years among medical students n: frequency; %: percentage; χ²: Chi-square test. p-values indicate the association between academic year and knowledge level (p < 0.05 considered statistically significant)

		Knowledge Level			Total	χ² (p-value)
Academic Year		Poor Knowledge	Adequate Knowledge	Good Knowledge		164.671 (<0.001)
Second	n	61	68	12	141	
	%	43.3%	40.4%	8.5%	100%	
Third	n	12	44	53	109	
	%	11.0%	40.4%	48.6%	100%	
Fourth	n	8	30	78	116	
	%	6.9%	25.9%	67.2%	100%	
Fifth	n	18	37	104	159	
	%	11.3%	23.3%	65.4%	100%	
Sixth	n	3	20	52	75	
	%	4.0%	26.7%	69.3%	100%	
Total	n	102	199	299	600	
	%	17.0%	33.2%	49.8%	100%	

Analysis of attitude scores and levels

Attitude scores of zero to six, seven to ten, and eleven to fourteen were classified as negative, neutral, and positive attitudes, respectively. The mean attitude score across all academic years was 9.83 ± 2.29. Analysis of attitude scores by academic year showed a statistically significant difference (F(4,595) = 10.883, p < 0.001). A Kruskal-Wallis test also demonstrated a significant difference in attitude scores across academic years (H(4) = 47.589, p < 0.001), with mean ranks increasing with academic progression. Responses to individual attitude questions are presented in Table 5. No statistically significant association was found between gender and attitude levels (χ²(2) = 2.048, p = 0.359). However, a Chi-square test assessing the association between academic year and attitude levels showed a statistically significant relationship (χ²(8) = 41.937, p < 0.001) (Table 6).

**Table 5:** Responses to attitude-related questions regarding antibiotic use and antimicrobial resistance among medical students n: frequency; %: percentage

Questions Evaluating Attitude	Agree		Disagree		Not Sure		Total
	n	%	n	%	n	%	n
You feel better after taking 2-3 doses of antibiotics	340	56.60%	65	10.70%	195	32.30%	600
Antibiotics help you recover if you have fever	283	47.10%	148	24.60%	169	28.10%	600
As a future physician, you will prescribe antibiotics if requested by a patient	83	13.80%	357	59.50%	160	26.70%	600
Prescribing antibiotics upon patient request causes no harm	57	9.50%	479	79.80%	64	10.70%	600
Unjustified antibiotics prescription burdens the Iraqi healthcare system	513	85.50%	31	5.20%	56	9.30%	600
Restricting antibiotic dispensing to prescription only is a good practice	538	89.60%	37	6.10%	25	4.10%	600
Iraq should adopt a prescription-only approach for antibiotics	492	82.00%	57	9.50%	51	8.50%	600

**Table 6:** Distribution of attitude levels toward antibiotic use and antimicrobial resistance across academic years among medical students n: frequency; %: percentage; χ²: Chi-square test. p-values indicate the association between academic year and attitude level (p < 0.05 considered statistically significant)

		Attitude Level			Total	χ² (p-value)
Academic Year		Negative Attitude	Neutral Attitude	Positive Attitude		41.937 (p < 0.001)
Second	n	24	89	28	141	
	%	17.00%	63.10%	19.90%	100%	
Third	n	10	46	53	109	
	%	9.20%	42.20%	48.60%	100%	
Fourth	n	6	55	55	116	
	%	5.20%	47.40%	47.40%	100%	
Fifth	n	10	72	77	159	
	%	6.30%	45.30%	48.40%	100%	
Sixth	n	4	37	34	75	
	%	5.30%	49.30%	45.30%	100%	
Total	n	54	299	247	600	
	%	9.00%	49.80%	41.20%	100%	

Analysis of practice scores and levels

Practice scores ranging from zero to five, six to eight, and nine to 12 were classified as poor, adequate, and good practice, respectively. The mean practice score was 3.37 ± 2.47. One-way ANOVA showed a statistically significant difference in practice scores across academic years (F(4,595) = 3.41, p = 0.009). A Kruskal-Wallis test confirmed this finding (H(4) = 14.254, p = 0.007). Practice levels differed by gender. Male students demonstrated higher proportions of adequate and good practice compared to female students, and this association was statistically significant (χ²(2, N = 597) = 9.06, p = 0.011). Responses to individual practice-related questions are presented in Tables 7, 8. Across all academic years, the majority of students demonstrated poor antibiotic use practices, while adequate practice was observed in a smaller proportion and good practice was rare (Table 9). The association between academic year and practice levels was not statistically significant (χ²(8, N = 600) = 9.18, p = 0.327). Mean knowledge, attitude, and practice scores by academic year are summarized in Table 10.

**Table 7:** Responses to practice-related questions regarding antibiotic use among medical students n: frequency; %: percentage

Questions Evaluating Practices	Yes		No		Sometimes	
	n	%	n	%	n	%
Do you consult a doctor before taking antibiotics?	117	19.50%	334	55.70%	149	24.80%
Do you give your antibiotics to family members if they have similar symptoms?	381	63.50%	111	18.50%	108	18.00%
Do you keep leftover antibiotics for future use?	433	72.20%	93	15.50%	74	12.30%
Do you stop antibiotics after feeling better without completing the course?	388	64.70%	128	21.30%	84	14.00%
Do you use antibiotics when you have a cold or the flu?	243	40.50%	162	27.0%	195	32.50%

**Table 8:** Sources of antibiotics reported by medical students n: frequency; %: percentage

Source of Antibiotics	Available at Home (n)	Available at Home (%)	Local Pharmacy (n)	Local Pharmacy (%)	Doctor (n)	Doctor (%)
From where do you obtain antibiotics?	254	42.30%	306	51.00%	40	6.70%

**Table 9:** Distribution of practice levels related to antibiotic use across academic years among medical students n: frequency; %: percentage; χ²: Chi-square test. p-values indicate the association between academic year and practice level (p < 0.05 considered statistically significant)

		Practice Level			Total	χ² (p-value)
Academic Year		Poor Practice	Adequate Practice	Good Practice		9.18 (p = 0.327)
Second	n	123	15	3	141	
	%	87.20%	10.60%	2.10%	100%	
Third	n	91	12	6	109	
	%	83.50%	11.00%	5.50%	100%	
Fourth	n	87	22	7	116	
	%	75.00%	19.00%	6.00%	100%	
Fifth	n	129	23	7	159	
	%	81.10%	14.50%	4.40%	100%	
Sixth	n	57	13	5	75	
	%	76.00%	17.30%	6.70%	100%	
Total	n	487	85	28	600	
	%	81.20%	14.20%	4.70%	100%	

**Table 10:** Mean with standard deviation of knowledge, attitude, and practice scores across academic years among medical students N: number of students; SD: standard deviation

Academic Year		Knowledge Score	Attitude Score	Practices Score
Second	Mean	2.6667	8.7660	2.8794
	N	141	141	141
	SD	1.36626	2.04464	2.24078
Third	Mean	4.0092	10.0642	3.1376
	N	109	109	109
	SD	1.22092	2.42394	2.51833
Fourth	Mean	4.4224	10.2672	3.8276
	N	116	116	116
	SD	0.99696	2.15632	2.68418
Fifth	Mean	4.3082	10.2201	3.4088
	N	159	159	159
	SD	1.19572	2.10980	2.38193
Sixth	Mean	4.5600	9.9867	3.8533
	N	75	75	75
	SD	0.85803	2.50671	2.48070
Total	Mean	3.9217	9.8300	3.3717
	N	600	600	600
	SD	1.36942	2.28834	2.46854

## Discussion

AMR is a public health crisis, and assessing awareness of this global problem is one of the important ways of combating it. To our knowledge, this is the first cross-sectional survey assessing the knowledge, attitudes, and practices of medical students regarding antibiotic use in southern Iraq. This study aimed to provide insight into the effectiveness of medical education in shaping practices regarding antibiotic use among future prescribers in the region.

Although 96% of final-year students correctly recognized that AMR is a global issue and 94.7% answered that unjustified use of antibiotics leads to AMR, only 6.7% demonstrated good practice levels, with the majority (76%) exhibiting poor antibiotic use practices. Specifically, 37.3% reported self-medicating with antibiotics for the common cold or influenza, 50.7% stopped taking antibiotics after feeling better without completing the prescribed course, and 68% stored antibiotics at home for future use. Moreover, 51% of all participants reported obtaining antibiotics directly from local pharmacies without a prescription, while only 6.7% obtained antibiotics using a prescription from a doctor. In Iraq, pharmacies are allowed to dispense a wide variety of antibiotics without a prescription according to Ministry of Health policies [7]. Systematic reviews from low- and middle-income settings show that weak enforcement of prescription laws and widespread availability of antibiotics over the counter are stronger determinants of misuse than individual knowledge or attitudes alone [20].

The study also revealed that despite improvement in knowledge levels and attitudes of medical students as they progressed through their medical education, practice patterns remained concerningly low, creating a gap between knowledge and practice levels. One possible explanation for this gap is the limited emphasis on practical antimicrobial stewardship teaching, with a greater focus on theoretical learning. Another contributing factor may be the persistence of cultural norms, as self-medication with antibiotics is common among the Iraqi population, as demonstrated in a 2021 cross-sectional study conducted among the general public in Baghdad [21]. Additionally, medical students may adopt practice habits by consulting senior staff before referring to clinical guidelines, as reported in a 2020 systematic review [26]. Inappropriate antibiotic use also remains prevalent among Iraqi physicians [27]. Similar gaps between knowledge of AMR and antibiotic use practices have been reported among medical students in other countries. A 2013 survey in China involving 1,236 students demonstrated a lack of correlation between knowledge levels and antibiotic-related behaviors [28]. Comparable findings have been reported in KAP studies from India [29], Nepal [30], and Jordan [31], which recommended better integration of practical AMR training within medical curricula.

Two additional findings emerged from the demographic analyses. First, a weak but statistically significant association was observed between having a family background in healthcare and attitude levels, with students reporting such a background demonstrating more negative attitudes toward antibiotic use. Although the association was weak, it may be explained by a tendency among educated individuals to make casual self-directed decisions, assuming sufficient knowledge, as suggested by a KAP study conducted among urban populations in Eritrea [32]. Second, the distribution of missing responses to the smoking question was observed more among female participants. Similar gender-related patterns have been reported in a study from Saudi Arabia, where female medical students demonstrated greater reluctance to report smoking behaviors due to cultural stigma and social pressures [33].

Based on these findings, revising medical school curricula to shift emphasis from theory-based learning to practice-oriented training in antimicrobial stewardship is warranted. This may include interactive teaching methods such as workshops, seminars, and hospital-based training. In addition, stricter policies are needed to address antibiotic misuse, including prohibiting the sale of antibiotics without a prescription and promoting adherence to updated clinical guidelines rather than reliance on informal advice from senior colleagues or community pharmacists.

Limitations

This study has several limitations. Its cross-sectional design precludes causal inference, and reliance on self-reported data introduces potential reporting bias. The questionnaire was not a standardized WHO KAP instrument, and the study was conducted at a single institution, which may limit generalizability. Exclusion of incomplete responses and voluntary participation may also have introduced selection bias. Despite these limitations, the large sample size and inclusion of students from a major medical college in southern Iraq enhance the reliability of the findings. Multicenter studies are recommended to validate these results and assess the impact of educational or policy interventions.

## Conclusions

Medical students at the College of Medicine, University of Basrah demonstrated poor antibiotic use practices, with no meaningful improvement across academic years, despite improvements in knowledge and attitudes. Self-medication was common, largely driven by unrestricted access to antibiotics through community pharmacies without a prescription. These findings highlight an urgent need for curriculum reform that prioritizes practical antimicrobial stewardship training and for stronger regulatory enforcement to limit non-prescription antibiotic sales. Such measures are essential to promote responsible antibiotic use among future physicians.

## Appendices

Appendix A. Questionnaire and scoring system

Study Title: Antibiotics and Their Use Among Medical Students at the University of Basra

Participation in this study is voluntary. You may withdraw at any time without any consequences.

This questionnaire is anonymous, and all responses will be kept confidential and used for research purposes only.

There are no right or wrong answers. Please respond honestly based on your knowledge and experience.

The questionnaire will take approximately five minutes to complete. By completing this questionnaire, you indicate your consent to participate

Section A: Demographic Information

Please select the option that best describes you

1. Gender                                   

Male                  Female

 2. Academic Year                      

Second              Third         Fourth          Fifth            Sixth

3. Do you smoke?                        

Yes            No

4. Is any member of your family a doctor or pharmacist?         

Yes          No

Section B: Knowledge

Please select the most appropriate answer for each question

5. What is the most common cause of colds and flu?      

Bacteria               Virus            Not sure

6. Antibiotics are effective against viruses.                                         

True             False              Not sure             

 7. The unnecessary use of antibiotics leads to bacterial resistance.    

True             False              Not sure         

 8. Using antibiotics without a medical reason can result in treatment failure in the future.

     True             False               Not sure

9. Bacterial resistance is an important global public health issue.         

True              False                Not sure

*Section C: Practices*  

Please answer the following questions based on your usual behavior by selecting the most appropriate option

10. Do you use antibiotics when you have a cold or the flu?                  

Yes          No       Sometimes

11. Where from do you obtain antibiotics?                              

a doctor        local pharmacy      Available at home

12. Do you consult a doctor before taking antibiotics?              

Yes               No             Sometimes

13. Do you stop taking the antibiotics after feeling better, without completing the course?

    Yes               No             Sometimes

 14. Do you keep leftover antibiotics for future use when you get sick again?    Yes               No             Sometimes

15. Do you give your antibiotics to a family member if they experience the same symptoms as you?

    Yes              No                Sometimes

Section D: Attitudes

Please indicate your level of agreement with the following statements

16. You feel better after taking 2-3 doses of antibiotics                  

Agree             Disagree             Not sure    

17. Antibiotics help you recover if you have fever.                        

Agree            Disagree                  Not sure

 18. After graduation, as a future physician, you will prescribe antibiotics if requested by a patient

    Agree         Disagree                 Not sure       

19. Prescribing antibiotics upon patient request causes no harm.      

Agree          Disagree                  Not sure

20. Unjustified antibiotics prescription burdens the Iraqi healthcare system   

    Agree           Disagree               Not sure

21. In most developed countries, antibiotics are only dispensed with a prescription. Restricting antibiotic dispensing to prescription- only is a good practice

    Agree         Disagree                 Not sure

22. Iraq should adopt a prescription-only approach for antibiotics.    

Agree        Disagree                Not sure

Scoring system

Knowledge Section (Questions 5-9)

Each correct answer was awarded 1 point, while incorrect and “Not sure” responses were awarded 0 points. The total knowledge score ranged from 0 to 5. Scores were categorized as poor knowledge (0-2 points), adequate knowledge (3-4 points), and good knowledge (5 points).

Practice Section (Questions 10-15)

Appropriate practices were awarded 2 points, while “Sometimes” responses were awarded 1 point and inappropriate practices were awarded 0 points. The total practice score ranged from 0 to 12. Scores of 0-5 were categorized as poor practice, 6-8 as adequate practice, and 9-12 as good practice.

Attitudes Section (Questions 16-22)

Positive attitudes toward appropriate antibiotic use were awarded 2 points, while “Not sure” responses were awarded 1 point and negative attitudes were awarded 0 points. The total attitude score ranged from 0 to 14. Scores were categorized as negative attitude (0-6), neutral attitude (7-10), and positive attitude (11-14).
